# Engaging Stakeholders in a Pragmatic Trial of Home-Delivered Meals for Persons with Dementia

**DOI:** 10.1093/geroni/igab046.3307

**Published:** 2021-12-17

**Authors:** Jill Harrison, Kathleen McAuliff, Kali Thomas

**Affiliations:** 1 Brown University, Brown University, Rhode Island, United States; 2 Brown University, Brown University/Providence, Rhode Island, United States

## Abstract

Gathering stakeholder feedback is essential to designing and implementing relevant and actionable research. Additionally, stakeholders, particularly those directly impacted by an intervention, bring unique insights and experiences. This paper presents the process and findings of a research endeavor to co-design a pragmatic clinical trial with a Stakeholder Advisory Panel (SAP) in an effort to understand facilitators and barriers to conducting the research and implementing study findings. The proposed trial compares the impact of frozen, drop-shipped meals versus daily home-delivered meals provided by Meals on Wheels (MOW) programs on the ability of individuals living with dementia to age in place. We recruited nine SAP members, who were compensated for their time. The SAP is composed of a) MOW clients with dementia, b) family members of MOW clients with dementia, c) paid or volunteer MOW drivers, and d) MOW staff. A research team member facilitated two 90-minute meetings with the SAP members via Zoom. The topics of the meetings included potential benefits and challenges with each mode of meal delivery, the importance of the primary outcome (time to nursing home placement), topics of interest to include in interviews with clients and caregivers, and how participants would explain the study to a friend. Audio of the Zoom meetings was transcribed, and meeting summaries were shared with the SAP. Benefits of forming and engaging a SAP, as well as key lessons learned from SAP members and how recommendations were reflected in changes to the study protocol will be discussed.

